# ‘I wouldn't push that further because I don't want to lose her’: a multiperspective qualitative study of behaviour change for long‐term conditions in primary care

**DOI:** 10.1111/hex.12304

**Published:** 2014-11-07

**Authors:** Cheryl Hunter, Carolyn A. Chew‐Graham, Susanne Langer, Jessica Drinkwater, Alexandra Stenhoff, Elspeth A. Guthrie, Peter Salmon

**Affiliations:** ^1^ Health Services Research Unit Nuffield Department of Population Health University of Oxford Oxford UK; ^2^ Research Institute, Primary Care and Health Sciences and National School for Primary Care Research Keele University Keele Staffs UK; ^3^ Department of Psychology Manchester Metropolitan University Manchester UK; ^4^ Leeds Institute of Health Sciences University of Leeds Leeds UK; ^5^ Institute of Psychology Health and Society University of Liverpool Liverpool UK; ^6^ Manchester Mental Health and Social Care Trust Manchester UK; ^7^ University of Manchester Manchester UK; ^8^ Department of Psychological Sciences University of Liverpool Liverpool UK

**Keywords:** behaviour change, chronic illness, health‐care professional–patient interaction, primary health care

## Abstract

**Background:**

Health outcomes for long‐term conditions (LTCs) can be improved by lifestyle, dietary and condition management‐related behaviour change. Primary care is an important setting for behaviour change work. Practitioners have identified barriers to this work, but there is little evidence examining practices of behaviour change in primary care consultations and how patients and practitioners perceive these practices.

**Objective:**

To examine how behaviour change is engaged with in primary care consultations for LTCs and investigate how behaviour change is perceived by patients and practitioners.

**Design:**

Multiperspective, longitudinal qualitative research involving six primary health‐care practices in England. Consultations between patients with LTCs and health‐care practitioners were audio‐recorded. Semi‐structured interviews were completed with patients and practitioners, using stimulated recall. Patients were re‐interviewed 3 months later. Framework analysis was applied to all data.

**Participants:**

Thirty‐two people with at least one LTC (chronic obstructive pulmonary disease, diabetes, asthma and coronary heart disease) and 10 practitioners.

**Results:**

Behaviour change talk in consultations was rare and, when it occurred, was characterized by deflection and diffidence on the part of practitioners. Patient motivation tended to be unaddressed. While practitioners positioned behaviour change work as outside their remit, patients felt uncertain about, yet responsible for, this work. Practitioners raised concerns that this work could damage other aspects of care, particularly the patient–practitioner relationship.

**Conclusion:**

Behaviour change work is often deflected or deferred by practitioners in consultations, who nevertheless vocalize support for its importance in interviews. This discrepancy between practitioners’ accounts and behaviours needs to be addressed within primary health‐care organizations.

## Background

Long‐term conditions (LTCs) are common in the general population, especially for people over the age of 50,^1^ and the number of people living with LTCs is expected to rise.^2^, ^3^ LTCs such as chronic obstructive pulmonary disease (COPD), coronary heart disease (CHD), diabetes and asthma currently affect between 1.7 and 6% of the general practice population in England.^4^ Effective management of the most prevalent LTCs requires patients to make dietary and lifestyle changes.^5^, ^6^, ^7^ Behaviour change, such as smoking cessation and increasing physical activity, can improve the prognosis of people with LTCs.^8^, ^9^, ^10^ Finding ways to support people with LTCs – and to promote behaviour change – is a key challenge facing health‐care services and governments globally.^11^, ^12^, ^13^

Primary care is considered the optimum health‐care setting to manage people with LTCs,^12^, ^14^ and LTC management accounts for a large proportion of the workload.^15^ Primary care is community‐based, accessible and able to offer continuity of care and of patient–practitioner relationships – key requirements for managing patient needs over time.^16^, ^17^ Several initiatives in the United Kingdom, such as the NHS and Social Care Long‐term Conditions Model^1^ and the Quality and Outcomes Framework (QOF),^18^ aim to improve quality of care and health outcomes for patients through system changes in primary care. As part of these system changes, there is a focus on patient self‐management, supported by initiatives such as the Expert Patient programme.^19^ The chronic care model suggests that 70–80% of people can self‐manage their conditions with support from health‐care services.^20^ This support includes information provision, lifestyle and dietary advice, and behaviour change interventions around issues such as smoking and alcohol use.

Despite evidence of, and a developing consensus regarding, effective behaviour change techniques for the use in primary care consultations^21^ and interventions,^22^ behaviour change work may be absent in practice.^23^ Barriers to behaviour change work in primary care have been identified. Practitioners cite lack of confidence and of knowledge about effective techniques as reasons for not engaging in this work.^23^, ^24^, ^25^ Also, practitioners mention a lack of time within consultations or describe patient characteristics such as lack of motivation, knowledge or ability as reasons for not including behaviour change work.^23^, ^25^, ^26^, ^27^ Blakeman *et al*.^28^ found that practitioners prioritized the patient–practitioner relationship over engaging in self‐management talk in consultations.

The aim of the current research was to investigate broadly the role of primary care consultations in the care of people with LTCs and their influence on patients’ health‐care use over time. Using audio‐recordings of consultations, and interviews with patients and practitioners, guided by stimulated recall, a multiperspective approach offered a way to gain insight into primary care practice alongside the perceptions of patients and practitioners.^29^ In this study, we added a longitudinal dimension with patient interviews at two time‐points, to investigate whether patients perceived the consultation as influencing health choices over time. This paper specifically examines one aspect of primary care consultations: practices of behaviour change. The aim of this paper was to investigate how practitioners and patients engaged in behaviour change in primary care consultations and how instances of behaviour change work were perceived by patients and practitioners.

## Methods

This study was part of an NIHR Programme Grant, CHOICE,^30^ and took place in North‐West England. Ethics approval was received from North West 8 Research Ethics Committee – GM East, 10/H1013/74.

### Design

This was a longitudinal qualitative study of primary care consultations for people with LTCs. A multiperspective approach was taken, combining consultation recordings and interview data from patients and practitioners.^31^

### Data collection

Primary care practices were invited to take part in the study by letter, email and/or phone. Using publicly available QOF data, practices with high prevalence rates in at least one of the four LTCs compared with the median for England were identified and approached, with the aim of achieving a sample with a diverse socioeconomic and geographic spread within the region of recruitment. Practice recruitment ceased when a sufficiently large and diverse sample of practices had agreed to participate. Researchers attended practices on agreed dates. We sampled for two types of consultations: disease reviews where the patient was invited to attend by the practice, and patient‐initiated appointments booked on or before the day of attendance. Relevant consultations were those that involved patients with one or more of four conditions: CHD, asthma, diabetes and COPD. Consultations were identified in two ways: either the consulting practitioner (general practitioner or practice nurse) was holding a chronic disease clinic for one or more of the included LTCs when the researcher attended and therefore all expected patients were eligible to participate; or the practitioner reviewed a general appointment list and selected all patients attending with one or more of the included conditions. On arrival at general practices, patients met reception staff who provided participant information sheets. A researcher then approached every patient to ask about participation.

We adopted a purposive sampling approach, aiming for maximum variation across conditions, age and gender of patients, and type of health‐care practitioner.

Patients and practitioners were interviewed separately after the recorded consultations, using a semi‐structured interview guide (Tables 1 and 2) and stimulated recall;^32^ short snippets of the consultation were played back during the interview to prompt thoughts and reflections. Snippets were identified using a consultation topic guide (see Table 3), and two of the researchers discussed which parts to focus on during stimulated recall, based on the on‐going analytical work. Practitioners were interviewed once about all consultations they recorded; patients were interviewed following their consultation and then invited to be followed up for a period of 3 months to gain insight into patients’ choices around health‐care use over time. Follow‐up with patients involved regular telephone contact and the completion of health‐care use diaries. Patients were then asked for a final interview at the end of 3 months.

**Table 1:** Initial topic guide headings for patients

First Interview People involved in care Identifying primary health‐care professional for condition/s Involvement of other health‐care professionals and their roles Decision making around different problems/exacerbations – past, present and future Self‐management Involvement of others (family/friends, etc.) Reviewing specific consultation (*using stimulated recall where relevant*) Reason/s for attending Initiation and purpose of consultation Expectations and hopes regarding consultation Any unmet needs/expectations and any issues not brought up Involvement in consultation and decision making Retrospective recall around content and value of consultation for self and practitioner Comparison with previous consultations Satisfaction or dissatisfaction with specific parts of consultation Examples of good/bad consultations Evaluation of consultation and any outcome/s from consultation Routine reviews Understanding of, and experience of, routine reviews Opinion/s on contribution of routine reviews to condition management Self‐management Management of condition by health‐care practitioners Unscheduled care Any recent use of unscheduled care What happened Any discussion of unscheduled care use with practitioners Any unmet needs or preferences around discussing unscheduled care use
Follow‐up Interview Review of health and health‐care use in last three months (*using telephone calls and health‐care logs to guide discussion*) Discussion of any exacerbations or problems in last three months Comparing and contrasting different services Experiences of and satisfaction with health‐care practitioners Routine reviews Understanding of, and experience of, routine reviews Opinion/s on contribution of routine reviews to condition management Self‐management Management of condition by health‐care practitioners Unscheduled care Any recent use of unscheduled care What happened Any discussion of unscheduled care use with practitioners Any unmet needs or preferences around discussing unscheduled care use

**Table 2:** Initial topic guide headings for health‐care practitioners

1 Management of LTCs within the practice Different roles within the practice Protocols around managing LTCs Goals of different types of LTC work 2 Consultations (*playing back snippets of consultations where relevant*) Type/s of consultation Purpose and value of consultations How consultations are organized Preparation for consultations Perspective on patient and practitioner expectations within specific consultations Perspective on patient and practitioner management of LTC/s, drawing on specific consultations Issues addressed in a specific consultation (and why) Issues not addressed or difficult to address in a consultation (and why) 3 Unscheduled care (*playing back snippets of consultations where relevant*) Discussion of unscheduled care in LTC consultations Role of primary care in reducing unscheduled care use

**Table 3:** Topic guide for analysing consultations for stimulated recall

Identify Context for consultation Focus of consultation Any additional issues brought up by patients in review appointments Outcome/s of consultation Discussion or mention of support at home (and by whom) Discussion or mention of mood (and by whom) Discussion or mention of self‐management (and by whom) Discussion or mention of exacerbations of condition/s (and by whom) Discussion or mention of unscheduled care use (and by whom) Any other issues arising that were not the primary/expected focus of the consultation
From these notes, identify prompts for the interview Specific to this consultation About consultations more generally
Identify time markers for sections of recording for stimulated recall

We adopted a two‐tiered approach to consent: patients gave initial written consent on the day to audio‐recording of their consultation and then were given up to 7 days to consider further participation and written consent to retention of the recordings. Where patients gave informed consent, their data were retained and analysed; however, full cases were prioritized in the multiperspective analysis presented below. Two patients’ data were excluded from the following analysis as their consultations dealt solely with an acute issue and included no discussion of LTCs.

### Analysis

All audio‐recordings (consultations and interviews) were transcribed verbatim and anonymized. Data were analysed using an integrative framework approach, adapting Ritchie and Spencer's approach^33^ for the incorporation of multiple perspectives on the same event (consultation). Specifically, the research team analysed transcripts inductively, comparing data within case and between case.^34^ The framework for the analysis was initially based on our analysis of the consultations, guided by pre‐determined topics of interest (Table 3). These topics were then tracked through the analysis of the first patient interview and practitioner interview for each case and summarized within case. These summaries shaped the telephone follow‐up calls and the follow‐up interview guide. Themes arising during analysis and data collection were discussed within the team and incorporated into further recruitment and topic guides and into the framework for analysis. Cases were re‐analysed as the framework developed over time. The research team was multidisciplinary, incorporating expertise in primary care, psychology and social anthropology. Data collection ended when the team were satisfied that analytical saturation was reached, with no new themes developing from analysis.

For the analysis, behaviour change topics included the following: commonly targeted health behaviours and specific self‐management behaviours known to affect LTCs. These were regarded as ‘behaviour change topics’ only if brought up in the context of reviewing patient behaviour related to one or more of the relevant LTCs. Mood management did not arise in this context; however, it arose in one consultation in relation to bereavement.^29^

## Results

### Sample characteristics

Thirty‐nine practices were approached and six participated in the study. Reasons given for non‐participation were as follows: involvement in other research and being too busy to participate, particularly because of QOF‐related activity. Across 6 primary care practices, 10 practitioners took part, including 5 general practitioners (GPs) and 5 practice nurses (PNs). Of 65 patients approached in practice waiting rooms, 34 were recruited into the study and agreed to audio‐recording of their consultations. Of those who declined participation, many did not give a reason, but some said that they were too ill or too busy to participate or that their consultation was not about their LTC. Patients were mainly White British (82.4%), 65% male, with ages ranging between 34 and 87. Most patients had more than one LTC (73.5%; 29.4% had at least two of COPD, CHD, asthma and diabetes) (see Table 4 for participant characteristics).

**Table 4:** Patient characteristics

Patient ID	Practice	Age	Gender	Condition/s
1	1	70	Male	COPD, cancer
2	1	62	Male	COPD, depression
3	1	51	Female	Asthma
4	2	46	Male	COPD
5	2	Not known	Female	Diabetes, COPD
6	2	85	Male	COPD, atrial fibrillation, dropped foot, balance problems
7	3	51	Male	Hypertension
8	3	Not known	Male	Diabetes
9	3	82	Male	Diabetes, asthma
10	3	54	Male	Diabetes, bowel problems
11	3	47	Female	Diabetes, cancer
12	3	87	Male	CHD, diabetes, depression
13	4	65	Female	Diabetes, COPD, angina
14	4	60	Male	Diabetes
15	4	Not known	Female	CHD, asthma
16	4	50	Female	Diabetes, nerve spasms
17	4	76	Female	CHD, cancer, high cholesterol
18	4	69	Female	COPD, arthritis
19^a^	4	74	Male	CHD, asthma, COPD, meningioma
20	4	50	Male	CHD, depression, blindness
21	4	43	Male	Diabetes
22	4	62	Male	CHD, diabetes, hypertension, CKD
23	4	58	Male	CHD, diabetes, cancer, piles
24	4	57	Female	Asthma, sarcoidosis, bronchiectasis
25^a^	4	Not known	Female	Diabetes
26	5	41	Female	Asthma
27	5	51	Male	Asthma, hypertension
28	5	73	Male	CHD, diabetes, gout
29	5	30s	Female	Asthma, depression, irritable bowel syndrome
30	5	76	Male	CHD, hypertension, arthritis, asbestosis
31	5	76	Female	Diabetes, arthritis
32	5	67	Male	Diabetes, hypertension, glaucoma
33	6	Not known	Male	Asthma, CHD
34	6	67	Male	CHD, hypertension

a Participants excluded from analysis as their consultations dealt solely with discrete acute issues and did not discuss LTCs (P19, a prostate exam; P25, pain due to neck injury).

Twenty‐nine consultations were audio‐recorded by practitioners, with the number recorded per practitioner varying between 1 and 8 (mean = 3). On 5 occasions, consultations were not recorded due to technical errors; in these instances, patients were still invited to take part in an interview about the consultation.

**Figure 1:**
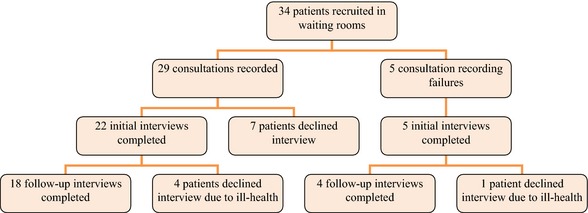
Recruitment and retention of patient participants.

There were 18 patients from whom consultation data, patient first interview, patient second interview and practitioner interview were all available – these constituted full cases. Of the remaining 16 patients, 4 declined participation in the follow‐up interview; 7 declined interviews but gave consent to retain their consultation; and 5 took part in either one or two interviews, but lacked a recorded consultation. Retention during follow‐up was high (81.5%), with 22 patients completing two interviews. Figure 1 details patients’ recruitment and retention.

In total, 84 transcripts were included in the analysis, generated from 32 patients and 10 practitioners, across six primary care practices.

Table 5 summarizes the behaviour change talk present in 27 consultations. Smoking, medication use and diet were the three most common behaviour change topics brought up in consultations as targets for behaviour change. Specific self‐management programmes or strategies, as means to address behaviour change, were only mentioned in four consultations; these included pulmonary or coronary rehabilitation programmes (*n* = 2) and rescue packs of antibiotics and steroids for COPD (*n* = 2).

**Table 5:** Behaviour change topics and responses

Behaviour change topics (*n* = no. of consultations where topic was mentioned)	Responses employed by practitioners^a^							
	No discussion – no need for behaviour change^b^	Gives information about behaviour and need for change	Explores triggers/motivation	Emphasizes importance of change	Directive advice	Suggests change	Gathers information about behaviour	Defers to another practitioner
Smoking (*n* = 10)	5	–	1	1	1	–	2	1
Diet (*n* = 9)	–	2	–	–	2	2	4	1
Medication use^c^ (*n* = 9)	–	2	–	–	5	–	–	–
Alcohol use (*n* = 6)	2	1	–	1	2	1	2	–
Exercise (*n* = 4)	–	1	1	1	–	–	2	–
Self–management strategies (*n* = 4)	–	3	–	1	1	–	–	–
Social activity (*n* = 1)	–	–	–	–	–	–	1	1

a Number of responses can be greater than number of consultations as, in some consultations, more than one strategy was employed, for example information gathered, then deferral to another practitioner.

b In these instances, patients either indicated that they did not engage in this behaviour, or the practitioner indicated that level of behaviour was satisfactory and did not need addressing.

c Medication use is defined as talk around how and when to use currently prescribed medications, rather than talk around new prescriptions.

### Findings

Two interlinked themes arose from the multiperspective analysis around behaviour change. The first theme, ‘Engaging in behaviour change talk’, drawing on all three sets of data, was that behaviour change talk was characterized by deflection and diffidence on the part of practitioners. Patient cues about motivation were often not followed up, and patients tended to remain unmotivated, uncertain or overwhelmed by the idea of behaviour change. The second theme, ‘Perceptions of behaviour change and the role of the practitioner’, drawing on the practitioners’ accounts, was that practitioners often positioned behaviour change work as beyond their scope or ability to achieve, and potentially damaging to other aspects of their work, notably the patient–practitioner relationship. Despite offering vocal support for behaviour change and self‐management, practitioners frequently minimized their ability and responsibility to focus on this work in practice.

In transcript extracts, [ ] indicates explanatory text and (…) indicates omitted text.

### Engaging in behaviour change talk

There were four primary behaviour change techniques employed by practitioners in consultations: advising change without discussion, giving or gathering information, tentative suggestions for patient to change and deferring an issue for discussion at a later point or with another practitioner. The use of these techniques fitted into one of two broad practitioner styles: diffident and deflecting. The diffident style was characterized by advising or suggesting change without discussion; sometimes, information‐gathering occurred before a withdrawal from discussion. The deflecting style was characterized by information‐gathering, followed by deferral of discussion. Both styles occasionally involved information‐giving, but this was characteristically one‐sided; that is, the practitioner gave information, but did not encourage discussion about this information. In only two instances, practitioners engaged with a motivational approach to behaviour change talk, where there was exploration of the patient's motivation, barriers and facilitators for change.

#### Diffident style

This style was reflected in practitioners advising or suggesting change, and then withdrawing from further discussion. On some occasions, the practitioner enquired into the detail of a behaviour, before shutting down this line of enquiry to move on to another, often biomedical matter. This withdrawal tended to occur at the first sign of reluctance or resistance from the patient. For instance, in the following extract with a female patient with asthma, the nurse asked the patient about stopping smoking:

PN1 Any thoughts of stopping at all?

P3 There's been lots of thoughts

PN1 Lots of thoughts, no action

P3 There's been lots of thought but there's no real action

PN1 Okay, you're just not ready to think about it at the moment?

P3 No, I don't, no, it's, some days yes, some days no, you know. You wake up with good intentions and then

PN1 I know

P3 something [annoys] you and you think ‘I need a cigarette’. And that starts you on for the rest of the day, the week, the month, the year, whatever

PN1 Well, whenever you feel that you're at that stage or you even want to think about it, you know you can come and talk about your options for helping you get through that?

In this segment, while the nurse started by asking about motivation (‘any thoughts?’), she closed down the discussion at the first sign of hesitancy. The patient offered one of her triggers for continuing smoking (‘something [annoys] you’), which is met by the nurse disengaging and deferring further discussion to a later time (‘whenever you feel that you're at that stage’). In interview, the PN indicated that she was trying to encourage the patient ‘just to think about quitting’, although it was evident that she had little confidence in her ability to do more:

PN1 A lot of people, don't want to change. Yes don't want to change. A lot of people will say that whatever normal, well it's the only vice I've got, or can't afford to buy this, or can't afford to buy that. So yes, financial and, yes reluctance to change.

Researcher And if someone's reluctant to change?

PN1 I think all you can do is just sow a little seed yourself, you know, little bits of information, offer them support for if they want to come back at any time

One GP used a similar technique of suggesting change and then withdrawing. His consultations were characterized by tentative language (‘you could try to decrease [alcohol intake]’ ‘[alcohol intake] could probably do with coming down a bit’) and negatively framed questions that closed down discussion (‘[smoking cessation] not something you want to consider at the moment?’). When asked about his approach in relation to one of his consultations, he offered the following justification:

GP1 If she wants to know more about [smoking cessation], that's fine. If she says ‘I don't want to deal with that now’, I'm not going to do any more about that, at that point (…) The success in smoking cessation is in people who want to stop

The effect of this style of communication is that the patients were left with a sense of personal responsibility for change, while uncertain about how to change and unclear about what help was available. When asked about how review consultations could help with her health, P3 predicated any hope for better health on her giving up smoking: ‘while you're smoking, [the asthma]'s never going to be any better is it? So maybe when you've given up smoking it might get possibly better’. At the same time, she saw herself as the main barrier to change: ‘The only thing [PN1] can't give me is the willpower [to stop smoking] (…) you're only going to do it when you think you're ready, or feel ready to do it’. Throughout the follow‐up period, the patient continued to smoke but wanted to stop for financial reasons. At follow‐up interview, she had decided to start Champix (a smoking cessation tablet available on prescription), as another nurse had informed her of its ‘good success rate’. In this case, the practice nurse's initial pessimism about the patient's desire to change and her own ability to enable change meant that the patient's motivations and options were unexplored until she saw another practitioner.

#### Deflecting style

This style sometimes overlapped with a diffident approach in that tentative language and negative phrasing were used as well as active deflection. Mostly, in this style of communication, practitioners gathered information about a patient's behaviour and then either deferred discussion to a later point or suggested that another practitioner was better equipped to deal with the issue. For instance, one nurse considered introducing a new self‐management strategy (rescue packs for COPD) following a discussion about recent exacerbations (in consultation with P4, a male patient with COPD: ‘I'm just wondering whether in the future we give you a small supply of antibiotics, then you can start on your own, but you'd still need to come and see us afterwards’, PN2). When the patient asked for more information, the nurse decided unilaterally to defer the discussion to the next review: ‘we'll go through that next time I think, I think we've done too much today’, PN2. This deflection seemed to serve the purpose of time management within the consultation, with the nurse deciding to prioritize other aspects of care when it appeared that the issue would require more discussion. It also contained an implicit judgement that the nurse should decide when a patient is ready to be introduced to new self‐management techniques.

Another form of deflection involved information‐gathering followed by implicit deferral to another practitioner. One GP used this technique twice: in the first instance, with a patient with diabetes who was struggling with his weight; in the second instance, with a patient who had recently started smoking again. On both occasions, despite cues from the patients that they were struggling with behaviour change (in consultation, P21 on diet, a male patient with diabetes: ‘I'd like to be better’, ‘I eat too much’, ‘I've got to try and manage it’) or despondent at the thought of change (in consultation, P23, a multimorbid male patient with CHD, diabetes and cancer, stated that regarding smoking: ‘I'm a lost cause’), the GP re‐directed the conversation back to biomedical matters. Deferring to another practitioner suggested that he was not able to help:

P23 I started smoking again though

GP3 Oh how many?

P23 I'm back to where I was before (…) a big pack of 50 g [tobacco] will last me a week, about 9 days, 8 or 9 days

GP3 So it's a lot though isn't it, I know you spoke to [healthcare assistant], she mentioned to you, didn't she, maybe did she?

P23 What?

GP3 About [healthcare assistant]'s talked to you about, did she talk to you about smoking or stopping smoking?

P23 I did stop for two months (…) I restarted again

GP3 But that's [healthcare assistant's] area of expertise, helping people to stop

P23 I think I'm a lost cause, if I've started again after that business

GP3 Yeah, and the other thing just to mention [GP starts talking about diabetes]

Here, the GP appeared reluctant to talk about smoking with the patient, despite the patient raising the issue. He deflects discussion by referring to the health‐care assistant repeatedly, giving the message that the GP is not the right person to tackle the issue, and disregarding the patient's assertion that he is a ‘lost cause’. The patient continued to smoke over the three month follow‐up period and was convinced that change was not achievable for him and that the practice could not help. In his follow‐up interview, he responded:

Researcher Do you still think there's nothing really that the practice can do?

P23 No. I think it's just a personal [trait], the way you are. It's difficult to change isn't it?

The GP described his tactic as ‘just trying to gauge his level of motivation to want to stop which I don't think was particularly high and he wasn't taking the bait really’, GP3. At the same time, the GP also indicated in his interview that ‘if they don't want to do it, no matter what a doctor says, they're not going to do it’, GP3, suggesting that he had little faith in his ability to influence patient motivation to change.

The deflecting style was characterized by the practitioner controlling the direction of the consultation and determining what topics could be discussed at that time or with them as practitioners. While the nurse and GP offered different reasons for changing direction (the nurse felt too much information had been covered; the GP felt another practitioner was better suited for the discussion), the overall effect is similar to that of the diffident style: the patient is left with a sense of personal responsibility for change, and the difficulties the patient faces with behaviour change are unexplored:

P21 I am overweight because it's my fault I'm overweight (…) [if I went to see another practitioner, as GP3 suggested] I find that I would be wasting, I feel like I was wasting that person's time, because I know what's wrong, I know what I should do

### Perceptions of behaviour change and the role of the practitioner

In interviews, practitioners agreed on the importance of self‐management and behaviour change for LTCs:

GP3 It's the lifestyle that really is the biggest determinant of health and ill‐health for diabetes and smoking and lung disease and so on

PN4 [Chronic disease management] is about self‐management

However, when it came to describing how behaviour change should work in practice, practitioners minimized their role with three different arguments.

#### Behaviour change is driven by patients, not by practitioners

First, practitioners consistently emphasized that behaviour change is the patient's responsibility and that behaviour change is dependent on people's motivation to change:

GP3 The driving factor, force, has got to be them [the patients]

PN5 [behaviour change has] got to come from them. You can intimidate somebody into stopping, or frighten somebody into stopping smoking. Yeah, they will do it but they don't sustain it, because they've been made to stop. They've got to want to do it

PN3 It's their health, their responsibilities

GP1 It's purely the success in smoking cessation is in people wanting to stop

Practitioners then positioned themselves as there to support the patient, if the patient wanted to change:

PN5 You're there to support them and be there for them

PN3 [my role]'s just keeping that contact with them as well, so that they know that they can turn to me if they've got a problem

Practitioners defined their role as supportive but passive and saw their responsibility as fulfilled by their offer of future support for patients.

#### Practitioners are limited in what they can do

When it came to discussing how practitioners can encourage or enable behaviour change, practitioners presented themselves as limited in the techniques they can use. They presented information‐giving and support as the techniques available to them and suggested that these techniques might influence patient motivation to change. Practitioners did not seem confident in the efficacy of these techniques:

GP2 If you can get them to help them to see the relationship [between lifestyle choices and their illnesses], then I think you're more likely hopefully for them to change their lifestyle

PN4 I see my role as education and support (…) understanding and trying to get, to empower them, to actually manage, it's their condition

PN1 I see my role specifically as, just passing on information that I know to hopefully help, that they will take on board to help manage their own symptoms

GPs saw information‐giving as an essential aspect of behaviour change work, but often raised doubts over what information alone could achieve:

GP3 I could say, ‘Here's an information leaflet or a little booklet’, that I did have on the shelf on the room, I know exactly where they are which says, ‘Diet and Diabetes’, I probably didn't do it because I didn't think it'd make any difference. I just, that kind of sense of despondency in some ways that giving him an information booklet I don't think is what he probably needs to make those changes

Some practitioners felt that, if they understood a patient's motivations and beliefs, they would be able to use this as a motivational lever to encourage or perhaps scare the patient into changing their behaviour.

GP3 You're gauging how interested people are and whether this, and just testing out whether this is the Achilles heel in his behaviour ‐ this is the information [that] strikes a chord with him that will actually get him to start to think and challenge his own habits, his own behaviour and whether we can sort of run with that

PN5 [I say to patients] ‘Do you want to be a grandparent whose children and grandchildren come and visit them, or do you want to be a grandparent who can take the grandchildren out? You're getting to that stage [of illness]’, and I kind of try and use some of those kinds of things so they can relate it to real life

However, these same practitioners used tentative language when talking about their ability to influence patients (‘gauging’, ‘testing’, ‘sort of run with that’; ‘kind of try and use some of those kinds of things’) and still talked about the need for patients to drive behaviour change, suggesting that they were not wholly confident that they could push patients towards a more motivated state.

#### The value of a good patient–practitioner relationship outweighs the value of challenging patient behaviour

The final argument that practitioners presented as a counter to their responsibility for behaviour change work centred around the patient–practitioner relationship. While practitioners agreed that behaviour change was important, they argued that the potential cost of challenging patient behaviour outweighed the benefits – it risked damaging the relationship without resulting in behaviour change:

PN5 The thing is you do not need to emphasise to a smoker this is bad for you. You don't need to go on and do this and do that ‐ they know that. And quite often if you do that they will shut down on you and they won't take it on board

GP1 I don't want [patients] to say I'm not going to see [the GP], because every time I go and see him, and we get this with people, he talks about smoking and I don't want to stop. And then they don't come back for other things, so you lose people if you are too evangelical, you lose people for other impacts you might have. So that's the reason for not banging on about it once she said she's not interested

Practitioners perceived themselves as limited in terms of what they could do to influence patient behaviour and therefore oriented themselves towards achieving what they know to be within their remit and expertise:

GP1 Our agenda is to rationalise their medication (…) to make sure they're to target on their medications, bloods, inhaler use if it's asthma (…) [by focusing on medication] we get QOF targets, the patients I think get their diseases reasonably well managed

This final line of reasoning suggests that some practitioners believed that achieving external system‐driven targets (such as QOF, the Quality and Outcomes Framework, which primary care practices are incentivized to achieve) ensured ‘good enough’ care for individual patients, given the constraints and limitations on what practitioners can do to change patient behaviour.

## Discussion

This study demonstrates a disjunction between how practitioners enact behaviour change in consultations and their expressed commitment to its importance for long‐term condition management. In the recorded consultations, any behaviour change talk evident was brief, diffident and limited, with no evidence of patient collaboration. This finding seemed consistent across consultations involving patients with multimorbidity or single morbidity. Practitioners typically employed two styles of behaviour change talk: a diffident, disengaged style, where they tentatively suggest change but withdraw from in‐depth discussion; and a deflecting style which moves behaviour change talk outside of the remit of the current consultation. Both styles effectively delegitimized behaviour change talk within the consultations, leaving patients unsure about the role of practitioners in aiding behaviour change.

In discussing behaviour change in interviews, practitioners emphasized its importance and its centrality in LTC management. They described a role for themselves in providing reminders and information to patients about the importance of behaviour change. However, they also emphasized constraints on their ability (and therefore, their responsibility) to effect behaviour change. Behaviour change work was constrained by a perceived lack of effective techniques to influence patient motivation, by the need to ensure a continued patient–practitioner relationship and, ultimately, by the patients themselves.^23^, ^25^, ^28^, ^35^ The relegation of behaviour change work to the responsibility of the patients or other practitioners offered a way for practitioners to disengage with this work in their consultations. In line with previous studies, this study highlights the infrequency of work around self‐management and behaviour change within primary care^28^ and adds further evidence of the uncertainty and reluctance on the part of practitioners to participate in this work.^23^, ^25^, ^35^ It builds on previous research by linking the types of communicative activity in consultations to both patient and practitioner attitudes towards behaviour change work.^28^, ^34^

Both patients and practitioners in this study viewed behaviour change work as the responsibility of the patient, and this in part could explain the absence of this work in consultations. This absence is likely to have a significant effect on patient engagement with change, as support from services and their social environment are important influences on patient attitudes towards, and ability to, self‐manage. Without supportive, collaborative relationships and support, it is likely that significant numbers of patients will continue to feel responsible for, yet unable to effect, behaviour change.^36^, ^37^, ^38^

There is a growing body of research demonstrating techniques that can result in behaviour change within primary care in the context of clinical trials, which may not require much time to implement.^39^, ^40^, ^41^, ^42^, ^43^ In addition, patients describe health practitioners as an important source of external motivation for self‐management.^44^ Yet successfully embedding behaviour change in practice, and motivating practitioners to change their consultation behaviour, is still elusive.

The practitioners in this study and in primary care in general are embedded in an increasingly busy and scrutinized context where certain tasks are valued and counted (like meeting biomedical targets) to the extent that care can become shaped around fulfilling these tasks,^45^ and others (such as less measurable behaviour change work) are not.^29^ It may be that practitioners feel both ill‐equipped to achieve behaviour change in patients and pressurized to achieve other targets, and the rhetoric of patient control and responsibility enabled them to legitimately distance themselves from this work.^46^ It is already known that training and education alone is insufficient to change practitioner behaviour.^47^ Practitioners are likely to only engage in behaviour change work if the structures in primary care promote and value this work.^17^, ^48^ This would mean a commitment to behaviour change as an activity beyond what is currently evidenced, and a cultural change in how practitioners view their role, and the role of the patient, in relation to behaviour change. Adapting routine consultations to run on a collaborative model of care may aid work around behaviour change if there is a commensurate shift in attitudes to a ‘joint responsibility’ ethos for achieving set goals.^49^ However, this shift is unlikely to occur unless collaborative working becomes the foundation through which other targets, such as QOF, can be achieved.

### Strengths and limitations

This study consisted of a relatively small sample of practitioners, which reflected difficulties in engaging general practices with the study. However, the sample size was similar to other studies adopting a multiperspective approach to analysis (between 8 and 17 practitioners),^28^, ^50^, ^51^ an approach which offers rich opportunities for small samples.^31^ The main reason practitioners gave for not engaging was that they had too many other demands on their time, notably QOF requirements; however, this was also a concern of practitioners who did participate. It is possible that practitioners who participated were more interested than others in psychological approaches and that non‐participating practitioners might give even less priority to behaviour change than those we studied. Nearly half of the patients declined participation when approached, and it is possible that these were more unwell than those who took part. Nevertheless, our sample included a broad range of LTCs and high levels of multimorbidity. In a study of this kind, where patients and practitioners are aware of being recorded, there is always the possibility that behaviours may have been changed as a result. To minimize this risk, the researchers were not present in the consultations.

Behaviour change was not initially an explicit focus of the study from which the present analysis was drawn, which aimed to explore chronic disease management within routine consultations. However, because data collection and analysis proceeded in parallel, we were able to incorporate discussion of behaviour change into respondent interviews early in the study when it emerged as a significant issue for practitioners and patients. Using stimulated recall in interviews enabled practitioners to reflect on their practice and facilitated recollection of particular patients and decisions over time. It also enabled discussion of behaviour change and its importance in relation to specific instances rather than generalized accounts.

A key strength of this study was that it combined analysis of practitioners’ and patients’ viewpoints with analysis of their consultations. This multiperspective approach offered a rich and nuanced way to explore practitioners’ attitudes and experiences of behaviour change alongside enactments of behaviour change in practice.

## Conclusion

Practitioners’ accounts endorsed the importance of behaviour change for LTCs; however, within primary care consultations, their approach to behaviour change tended to be diffident and limited, with little direct challenge to, or exploration of, patient motivations and beliefs. Practitioners employed three minimizing strategies within their interview accounts, which had the effect of absolving practitioners of responsibility for, and reinforcing current practice around, behaviour change. These strategies positioned patients as responsible for any failure to pursue or achieve behaviour change. In addition, these strategies reinforced the practitioners’ control of the content or agenda of consultations in which behaviour change could be dealt with. This in effect left patients feeling personally responsible yet unconfident and unclear about the role of primary care in assisting behaviour change.

It is likely that change in how practitioners engage with patients will only be achieved through an extensive cultural and attitudinal shift within primary care. Collaborative care planning may be one model that could enable behaviour change work within primary care, but to be adopted successfully, this model would need to be accompanied by a change in the attitudes and culture of primary care.

## Disclaimer

This article presents independent research funded by the National Institute for Health Research (NIHR) under its Programme Grants for Applied Research scheme (RP‐PG‐0707‐10162). The views expressed are those of the authors and not necessarily those of the NHS, the NIHR or the Department of Health.

## References

[1] Department of Health . Supporting People With Long Term Conditions: An NHS and Social Care Model to Support Local Innovation and Integration. London: Department of Health, 2005.

[2] Mathers CD , Loncar D . Projections of global mortality and burden of disease from 2002 to 2030. PLoS Medicine, 2006; 3: e442. 17132052 10.1371/journal.pmed.0030442 PMC1664601

[3] World Health Organisation . Global Status Report on Non‐Communicable Diseases 2010. Geneva: World Health Organisation, 2011.

[4] Prescribing and Primary Care Team . Quality and Outcomes Framework: Achievement, Prevalence and Exceptions Data, 2012/13. London: Health and Social Care Information Centre, 2013.

[5] Ory MG , Jordan PJ , Bazzarre T . The Behavior Change Consortium: setting the stage for a new century of health behavior‐change research. Health Education Research, 2002; 17: 500–511. 12408195 10.1093/her/17.5.500

[6] McGinnis JM , Williams‐Russo P , Knickman JR . The case for more active policy attention to health promotion. Health Affairs, 2002; 21: 78–93. 10.1377/hlthaff.21.2.78 11900188

[7] Blank L , Grimsley M , Goyder E , Ellis E , Peters J . Community‐based lifestyle interventions: changing behaviour and improving health. Journal of Public Health, 2007; 29: 236–245. 17601764 10.1093/pubmed/fdm041

[8] Critchley JA , Capewell S . Mortality risk reduction associated with smoking cessation in patients with coronary heart disease: a systematic review. Journal of the American Medical Association, 2003; 290: 86–97. 12837716 10.1001/jama.290.1.86

[9] Warburton DER , Nicol CW , Bredin SSD . Health benefits of physical activity: the evidence. Canadian Medical Association Journal, 2006; 174: 801–809. 16534088 10.1503/cmaj.051351 PMC1402378

[10] Willemse BWM , Postma DS , Timens W , ten Hacken NHT . The impact of smoking cessation on respiratory symptoms, lung function, airway hyperresponsiveness and inflammation. European Respiratory Journal, 2004; 23: 464–476. 15065840 10.1183/09031936.04.00012704

[11] Boyce T , Robertson R , Dixon A . Commissioning and Behaviour Change: Kicking Bad Habits Final Report. London: The King's Fund, 2008.

[12] World Health Organisation . The World Health Report 2008: Primary Healthcare: Now More Than Ever. Geneva: World Health Organisation, 2008.

[13] US Department of Health and Human Services . Multiple Chronic Conditions – A Strategic Framework: Optimum Health and Quality of Life for Individuals With Multiple Chronic Conditions. Washington, DC: US Department of Health and Human Services, 2010.

[14] Bodenheimer T . The future of primary care: transforming practice. New England Journal of Medicine, 2008; 359: 2086–2089. 10.1056/NEJMp0805631 19005190

[15] Department of Health . Long Term Conditions Compendium of Information, 3rd edn London: Department of Health, 2012.

[16] Starfield B . Is primary care essential? The Lancet, 1994; 344: 1129–1133. 10.1016/s0140-6736(94)90634-3 7934497

[17] Kennedy A , Bower P , Reeves D *et al* Implementation of self management support for long term conditions in routine primary care settings: cluster randomised controlled trial. British Medical Journal, 2013; 346: f2882. 23670660 10.1136/bmj.f2882 PMC3652644

[18] Department of Health . Investing in General Practice: The new General Medical Services Contract. London: Department of Health, 2003.

[19] Department of Health . The Expert Patient: A New Approach to Chronic Disease Self‐Management for the 21st Century. London: Department of Health, 2001.

[20] Wagner EH , Austin BT , Davis C , Hindmarsh M , Schaefer J , Bonomi A . Improving chronic illness care: translating evidence into action. Health Affairs, 2001; 20: 64–78. 11816692 10.1377/hlthaff.20.6.64

[21] Noordman J , van der Weijden T , van Dulmen S . Communication‐related behavior change techniques used in face‐to‐face lifestyle interventions in primary care: a systematic review of the literature. Patient Education and Counseling, 2012; 89: 227–244. 22878028 10.1016/j.pec.2012.07.006

[22] Cane J , Richardson M , Johnston M , Ladha R , Michie S . From lists of behaviour change techniques (BCTs) to structured hierarchies: comparison of two methods of developing a hierarchy of BCTs. British Journal of Health Psychology, 2014. doi: 10.1111/bjhp.12102. 10.1111/bjhp.12102 24815766

[23] Chisholm A , Hart J , Lam V , Peters S . Current challenges of behavior change talk for medical professionals and trainees. Patient Education and Counseling, 2012; 87: 389–394. 22205055 10.1016/j.pec.2011.12.001

[24] Laws RA , Kemp LA , Harris MF , Powell Davies G , Williams AM , Eames‐Brown R . An exploration of how clinician attitudes and beliefs influence the implementation of lifestyle risk factor management in primary healthcare: a grounded theory study. Implementation Science, 2009; 4: 66. 19825189 10.1186/1748-5908-4-66 PMC2770564

[25] Taylor CA , Shaw RL , Dale J , French DP . Enhancing delivery of health behaviour change interventions in primary care: a meta‐synthesis of views and experiences of primary care nurses. Patient Education and Counseling, 2011; 85: 315–322. 21051174 10.1016/j.pec.2010.10.001

[26] Elwell L , Povey R , Grogan S , Allen C , Prestwich A . Patients’ and practitioners’ views on health behaviour change: a qualitative study. Psychology and Health, 2013; 28: 6, 653–674. 10.1080/08870446.2012.744008 23278305

[27] MacGregor K , Handley M , Wong S *et al* Behavior‐Change Action Plans in Primary Care: a feasibility study of clinicians. Journal of the American Board of Family Medicine, 2006; 19: 215–223. 16672674 10.3122/jabfm.19.3.215

[28] Blakeman T , Bower P , Reeves D , Chew‐Graham C . Bringing self‐management into clinical view: a qualitative study of long‐term condition management in primary care consultations. Chronic Illness, 2010; 6: 136–150. 20444765 10.1177/1742395309358333

[29] Chew‐Graham C , Hunter C , Langer S *et al* How QOF is shaping primary care review consultations: a longitudinal qualitative study. BMC Family Practice, 2013; 14: 103. 23870537 10.1186/1471-2296-14-103 PMC3726490

[30] Manchester Mental Health and Social Care Trust CHOICE Project. Available at: http://choice.mhsc.nhs.uk/, accessed 30 April 2013.

[31] Kendall M , Murray SA , Carduff E *et al* Use of multiperspective qualitative interviews to understand patients’ and carers’ beliefs, experiences, and needs. British Medical Journal, 2009; 339: b4122. 19828645 10.1136/bmj.b4122

[32] Veldhuijzen W , Mogendorff K , Ram P , van der Weijden T , Elwyn G , van der Vleuten C . How doctors move from generic goals to specific communicative behavior in real practice consultations. Patient Education and Counseling, 2013; 90: 170–176. 23218241 10.1016/j.pec.2012.10.005

[33] Ritchie J , Spencer L . Qualitative data analysis for applied policy research In Bryman A, Burgess RG (eds) Analyzing Qualitative Data. London: Routledge, 1994: 173–194.

[34] Salmon P , Mendick N , Young B . Integrative qualitative communication analysis of consultation and patient and practitioner perspectives: towards a theory of authentic caring in clinical relationships. Patient Education and Counseling, 2011; 82: 448–454. 21111558 10.1016/j.pec.2010.10.017

[35] Chew‐Graham CA , May CR , Roland MO . The harmful consequences of elevating the doctor‐patient relationship to be a primary goal of the general practice consultation. Family Practice, 2004; 21: 229–231. 15128679 10.1093/fampra/cmh301

[36] Gately C , Rogers A , Sanders C . Re‐thinking the relationship between long‐term condition self‐management education and the utilisation of health services. Social Science & Medicine, 2007; 65: 934–945. 17521790 10.1016/j.socscimed.2007.04.018

[37] Greenhalgh T . Patient and public involvement in chronic illness: beyond the expert patient. British Medical Journal, 2009; 338: b49. 19223339 10.1136/bmj.b49

[38] Lawn S , McMillan J , Pulvirenti M . Chronic condition self‐management: expectations of responsibility. Patient Education and Counseling, 2011; 84: e5–e8. 20705412 10.1016/j.pec.2010.07.008

[39] Whitlock EP . Behavioral counseling interventions in primary care to reduce risky/harmful alcohol use by adults: a summary of the evidence for the US Preventive Services Task Force. Annals of Internal Medicine, 2004; 140: 557–568. 15068985 10.7326/0003-4819-140-7-200404060-00017

[40] Rubak S , Sandbaek A , Lauritzen T , Christensen B . Motivational interviewing: a systematic review and meta‐analysis. British Journal of General Practice, 2005; 55 : 305–312. 15826439 PMC1463134

[41] Stange KC , Woolf SH , Gjeltema K . One minute for prevention: the power of leveraging to fulfill the promise of health behavior counseling. American Journal of Preventive Medicine, 2002; 22: 320–323. 11988386 10.1016/s0749-3797(02)00413-0

[42] Rice VH , Stead LF . Nursing interventions for smoking cessation. Cochrane Database of Systematic Reviews, 2008; 1: CD001188. 10.1002/14651858.CD001188.pub3 18253987

[43] Kulick D , Langer RD , Ashley JM , Gans KM , Schlauch K , Feller C . Live well: a practical and effective low‐intensity dietary counselling intervention for use in primary care patients with dyslipidemia – a randomized controlled pilot trial. BMC Family Practice, 2013; 14: 59. 23663789 10.1186/1471-2296-14-59 PMC3662581

[44] Jowsey T , Pearce‐Brown C , Douglas K , Yen L . What motivates Australian health service users with chronic illness to engage in self‐management behaviour? Health Expectations, 2014; 17: 266–276. 10.1111/j.1369-7625.2011.00744.x PMC5060713 22070529

[45] Swinglehurst D , Greenhalgh T , Roberts C . Computer templates in chronic disease management: ethnographic case study in general practice. BMJ Open, 2012; 2: e001754. 10.1136/bmjopen-2012-001754 PMC3533031 23192245

[46] Salmon P , Hall GM . Patient empowerment and control: a psychological discourse in the service of medicine. Social Science and Medicine, 2003; 57: 1969–1980. 14499519 10.1016/s0277-9536(03)00063-7

[47] Grimshaw JM , Shirran L , Thomas R *et al* Changing Provider Behavior: an Overview of Systematic Reviews of Interventions. Medical Care, 2001; 39 : II2–II45. 11583120

[48] Salmon P , Peters S , Rogers A *et al* Peering through the barriers in GPs’ explanations for declining to participate in research: the role of professional autonomy and the economy of time. Family Practice, 2007; 24: 269–275. 17504773 10.1093/fampra/cmm015

[49] Coulter A , Roberts S , Dixon A . Delivering Better Services for People With Long‐Term Conditions: Building the House of Care. London: King's Fund, 2013.

[50] Cape J , Geyer C , Barker C *et al* Facilitating understanding of mental health problems in GP consultations: a qualitative study using taped‐assisted recall. British Journal of General Practice, 2010; 60: 837–845. 20939945 10.3399/bjgp10X532567 PMC2965968

[51] Mendick N , Young B , Holcombe C , Salmon P . The ethics of responsibility and ownership in decision‐making about treatment for breast cancer: triangulation of consultation with patient and surgeon perspectives. Social Science & Medicine, 2010; 70: 1904–1911. 20382463 10.1016/j.socscimed.2009.12.039

